# Aortic Relative Pressure Components Derived from Four-Dimensional Flow Cardiovascular Magnetic Resonance

**DOI:** 10.1002/mrm.25015

**Published:** 2013-11-18

**Authors:** Pablo Lamata, Alex Pitcher, Sebastian Krittian, David Nordsletten, Malenka M Bissell, Thomas Cassar, Alex J Barker, Michael Markl, Stefan Neubauer, Nicolas P Smith

**Affiliations:** 1 Department of Biomedical engineering, Division of Imaging Sciences, The Rayne Institute, Kings College School of MedicineUnited Kingdom; 2 Department of Computer Science, University of OxfordUnited Kingdom; 3 Oxford Centre for Clinical Magnetic Resonance Research, Radcliffe Department of Medicine, Division of Cardiovascular Medicine, University of OxfordUnited Kingdom; 4 Departments of Radiology and Biomedical Engineering, Northwestern University Feinberg School of MedicineChicago, Illinois, USA

**Keywords:** aorta, cardiac magnetic resonance imaging, blood pressure, hemodynamics, noninvasive pressure estimation

## Abstract

**Purpose:**

To describe the assessment of the spatiotemporal distribution of relative aortic pressure quantifying the magnitude of its three major components.

**Methods:**

Nine healthy volunteers and three patients with aortic disease (bicuspid aortic valve, dissection, and Marfan syndrome) underwent 4D-flow CMR. Spatiotemporal pressure maps were computed from the CMR flow fields solving the pressure Poisson equation. The individual components of pressure were separated into time-varying inertial (“transient”), spatially varying inertial (“convective”), and viscous components.

**Results:**

Relative aortic pressure is primarily caused by transient effects followed by the convective and small viscous contributions (64.5, 13.6, and 0.3 mmHg/m, respectively, in healthy subjects), although regional analysis revealed prevalent convective effects in specific contexts, e.g., Sinus of Valsalva and aortic arch at instants of peak velocity. Patients showed differences in peak transient values and duration, and localized abrupt convective changes explained by abnormalities in aortic geometry, including the presence of an aneurysm, a pseudo-coarctation, the inlet of a dissection, or by complex flow patterns.

**Conclusion:**

The evaluation of the three components of relative pressure enables the quantification of mechanistic information for understanding and stratifying aortic disease, with potential future implications for guiding therapy. Magn Reson Med 72:1162–1169, 2014. © 2013 The Authors. Magnetic Resonance in Medicine published by Wiley Periodicals, Inc. on behalf of International Society for Magnetic Resonance in Medicine. This is an open access article under the terms of the Creative Commons Attribution License, which permits use, distribution and reproduction in any medium, provided the original work is properly cited.

## INTRODUCTION

Aortic aneurysm is a common cause of morbidity and mortality [1]. In this disease, the spatial distribution and temporal changes in aortic pressure play an important role in driving progression of dilation, and are critical in initiating complications such as dissection and rupture. Understanding the nature of these pressure changes has significant potential to provide new insights into the mechanisms of aneurysm growth and related complications, to enable novel approaches for stratifying patients, and to inform the development and selection of therapies for patients at risk.

The Navier-Stokes equation describes blood pressure as a consequence of two forces acting upon the fluid: inertial and viscous forces. The inertial force, producing acceleration of the flow, can itself be resolved into two further components: one that causes the temporal acceleration at a fixed point in space (*transient* acceleration), and one that causes the spatial acceleration at a fixed time point (*convective* acceleration). Viscous force represents tractions that arise as a result of friction. Dynamic pressure (not to be confused with total pressure defined as static plus dynamic used in catheter measurements) can, therefore, be decomposed in three components, transient, convective and viscous, and their independent analysis has the potential to enhance our understanding of aortic disease processes. Our hypothesis is that each of these pressure components reveals independent characteristics that relate to the performance of the central circulatory system, specifically: the transient component describes the interaction between cardiac pump action and aortic compliance; the convective component captures the effects of vessel geometry (tortuosity, stenosis, or tapering); and the viscous component quantifies inefficiencies due to friction.

Computation of pressure components has been previously performed mainly for the analysis of ventricular flow using two-dimensional (2D) data. These analyses, typically of transient (local inertial) and convective components in 2D MRI slices, were fundamental for the development of single slice fast measurement [2]. The relative contribution of convective and transient effects has also been analyzed using Doppler ultrasound to characterize diastolic filling function, and the convective deceleration load is described as an important determinant of ventricular inflow [3]. However, to our knowledge, no comprehensive description of the three pressure components in the aorta has been reported.

Phased-contrast MRI (PC-MRI) has emerged over recent years as a valuable approach for the comprehensive visualization and quantification of blood flow within a large 3D volume of interest, such as the entire thoracic aorta [4,5]. Flow patterns derived from this modality have been shown to vary with anatomical location and extent across a range of cardiac and vascular diseases [5]. We have recently described and verified a method for the noninvasive estimation of pressure based on 4D flow data using a finite-element solution [6], which has several advantages over previous methods [7–13]. This technique underpins the specific aims of this work: (i) to demonstrate the feasibility and potential utility of analyzing the three components of relative aortic pressure, and (ii) to describe the pressure (component) distribution in healthy volunteers, comparing them to three selected patients with aortic disease. A preliminary version of this work appeared in a conference abstract [14].

## METHODS

**FIG 1:**
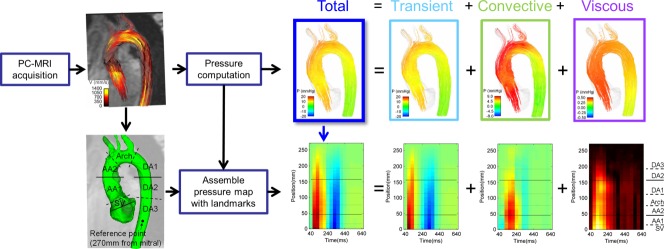
Methodology for the computation of the spatiotemporal maps of pressure in the aorta. The top of the illustration represents the data workflow from a single frame, frame 4, which constitutes the fourth column in the spatiotemporal map, as indicated by the blue arrow. The horizontal lines in the spatiotemporal map correspond to specific plane locations in the aorta, as illustrated in detail in Figure 2. [Color figure can be viewed in the online issue, which is available at http://wileyonlinelibrary.com.]

Calculation of relative pressure is performed by solving the Navier Stokes equation over PC-MRI velocity data. Individual time frames are assembled to produce *spatiotemporal relative pressure maps* (see Figure 1). Relative pressure here refers to values computed *relative* to a reference point in the vessel lumen (see Supplemental Figure S1, which is available online).

### Case Selection

All subjects gave written informed consent, and all study-related procedures were approved by a research ethics committee and in accordance with guidelines by the ICH-GCP. A description of volunteers and patients (bicuspid aortic valve, BAV; aortic dissection, AoD; Marfan syndrome, MFS) is provided in Table 1.

**Table 1:** Characteristics of Volunteers and Patients of the Study

Case	Characteristics
Volunteers	N = 9. Ages: 25 to 30
BAV case	55 years, male Has a bicuspid aortic valve (without stenosis, peak blood velocity of 1.9m/s) and an aneurysm of the ascending aorta.
AoD case	39 years, male Sustained a type A aortic dissection two years previously, for which he underwent immediate aortic valve and aortic root replacement with a composite mechanical valved aortic conduit. The dissected distal ascending aorta and dissected aortic arch were not treated surgically, giving rise to a “double barrelled” distal ascending aorta and arch. The false lumen re-enters the true lumen at the proximal descending aorta.
MFS case	49 years, female Marfan syndrome, no prior surgical treatment and was treated with beta-blockers. B-blocking agents were discontinued in patients 72 hours prior to data acquisition.

### Velocity Data Acquisition

The approach used is that of Markl and co-workers [15]. A 3D volume of interest covering the aorta from the left ventricular outflow tract to the diaphragm was prescribed. Acquisition characteristics are provided in Table 2.

**Table 2:** Acquisition Parameters of PC-MRI Sequences

Machine	3 Tesla MR system (Trio, Siemens AG, Erlangen, Germany)
Coil	standard 32-channel phased-array coil
Sequence	k-space segmented 3D RF-spoiled gradient-echo sequence with interleaved three-directional velocity encoding
Gating	Respiratory navigator gating and prospective ECG gating
Spatial resolution	1.25–1.77 x 1.25–1.77 x 3.2mm^3^
Matrix size	192–256 x 120–192
Field of view	Rectangular: 320–340 to 200–256) mm^2^
Number of slices	20–40
Temporal resolution	40ms
Velocity encoding	1.5 m/s
Frames per cardiac cycle	16–20
Flip angle	7°
TR	5ms
TE	2.519ms
Segmentation factor	2

### CMR Data Preprocessing

Data preprocessing was performed to correct for noise, eddy currents, and velocity aliasing to generate a reliable flow field as previously described [9]. The fluid domain over which calculations were performed was defined by semi-automatic segmentation of the aorta using ITK-Snap [16] from an image representing the average velocity magnitude.

### Pressure Estimation

The computational method of estimation of relative pressure is based on a finite-element approach [6]. In brief, for an incompressible, laminar Newtonian fluid of density *ρ* and viscosity *μ*, the relationship between pressure and flow can be described using the Navier-Stokes equation:


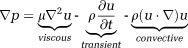


where *p* is pressure, *μ* is blood velocity, and *t* is time, and where the three components of pressure are described. In the equation above the body force has been removed because it has no effect on the flow, as the buoyancy force cancels out the gravitation force [17], and any effect of magnetic fields on flow measurements are neglected.

Pressure is computed by taking the divergence of the Navier-Stokes equation and solving the resulting pressure Poisson equation (PPE) with *ρ* = 1.06 x 10^3^ Kg/m^3^ and *μ* = 0.0035 Pa⋅s, values taken from [9,17,18]. The contribution that each component makes to overall relative pressure was computed by solving the PPE equation with each of the fluid forces independently. The reference point for the computation of relative pressure is fixed at the minimum total anatomical length for all cases under study, at 270 mm from the aortic valve plane.

### Calculation of Spatiotemporal Maps of Relative Pressure

Average pressure values are computed alongside the centerline of the aorta to conform the maps of relative pressure. Centerline was obtained using a skeletonization algorithm [19] from the Gerardus project (http://code.google.com/p/gerardus). A set of planes were prescribed perpendicular to the centerline at evenly spaced points separated by 1mm. Each plane defined a set of locations where pressure values were linearly interpolated. For each frame, the average value of pressure was calculated at each plane, conforming the map of relative pressure.

### Regional Analysis by Normalization to Length: Average Pressure Gradient

**FIG 2:**
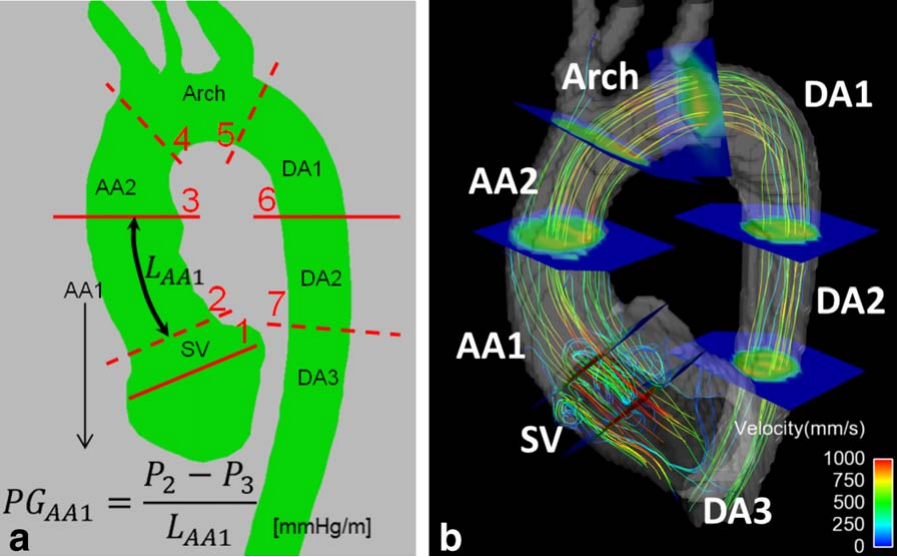
a: Definition of the seven aortic anatomical regions (Sinus of Valsalva – SV; ascending aorta – AA1 and AA2; arch; and descending aorta – DA1, DA2, DA3) and their average pressure gradient (PG): the pressure difference between bounding planes (planes numbered 1 to 7) is divided by the length of the aortic segment of the region (L_AA1_ in the example illustrated). b: Illustration of the result of the placement of bounding planes in one case, highlighting the vortical flow at SV that is used to define planes 1 and 2. [Color figure can be viewed in the online issue, which is available at http://wileyonlinelibrary.com.]

Regional analysis was performed by dividing the aorta in seven regions by manually placing seven planes using EnSight (CEI, Apex, NC). Aortic valve plane (plane 1) and sinotubular junction (plane 2) were placed after visualization of vortical flows at the Sinus of Valsalva (SV), see Figure 2. Comparison between subjects is enabled by normalization to length (see Figure 2) of the region, computing thus an *average pressure gradient*.

## RESULTS

**FIG 3:**
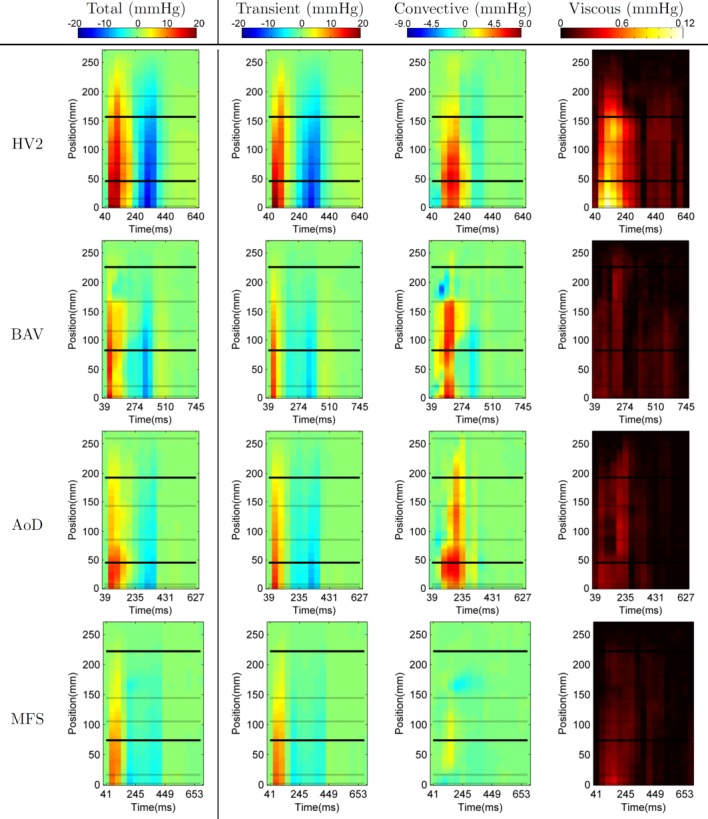
Spatiotemporal maps of relative pressure in the one representative healthy volunteer (HV2) and the three patients, showing the average pressure alongside the length of the aorta (Y axis) through time (X axis). Horizontal lines correspond to the location of the planes dividing the anatomical regions in the aorta (see Figure 2). Note that the scale of each pressure component is different.

The spatiotemporal relative pressure maps are shown in Figure 3 as a visualization of aortic relative pressure along the aorta. Description of flow characteristics and illustration of average pressure gradients are provided in Supplemental Figures S2 and S3, respectively.

### Healthy Volunteers

In this cohort, relative pressure is primarily caused by transient effects (14.1 mmHg or 64.5 mmHg/m at time of peak temporal acceleration), followed by the convective (2.9 mmHg or 13.6 mmHg/m at time of peak velocity) and a small viscous contribution (0.08 mmHg or 0.3 mmHg/m also at time of peak velocity, see Figure 5). Nevertheless, convective component can be of the same or even larger magnitude when compared with the transient at specific locations and times (see the arch at peak velocity, sixth frame.

**FIG 4:**
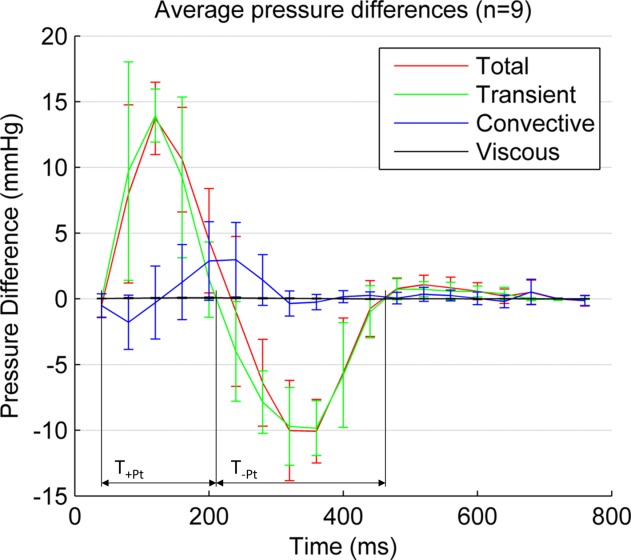
Average temporal evolution of aortic relative pressure between the aortic valve plane (plane 1 in Figure 2) and the descending aorta (at a distance of 270 mm) in the nine healthy volunteers, decomposed in its three components: transient, convective and viscous. Confidence intervals indicate mean ± 1 std. Peak value of the transient component (14.1 mmHg) occurs at the instant of peak acceleration, and peak values of convective (2.9 mmHg) and viscous (0.08 mmHg) at the instant of peak velocity (acceleration and velocity magnitude transients are not shown for better clarity). +T_Pt_ and −T_Pt_ are the duration of the positive and negative two phases of the transient component of pressure. [Color figure can be viewed in the online issue, which is available at http://wileyonlinelibrary.com.]

**FIG 5:**
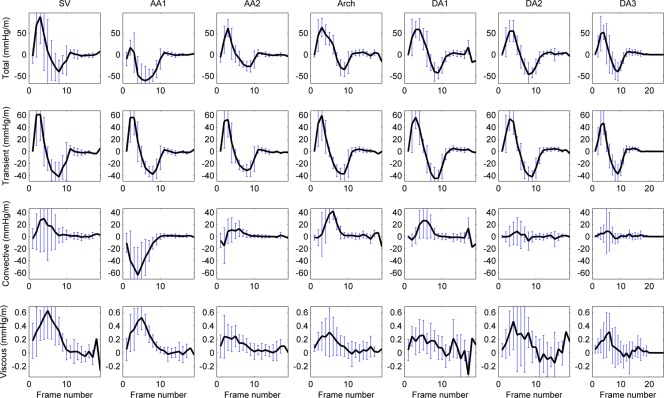
Average temporal profile of the pressure gradient in healthy subjects (n = 9) and its components in the seven anatomical regions. A temporal shift of the transient component can be appreciated (see how peak positive and negative values are delayed alongside the aorta). Negative values of the convective component at AA1 are explained by the expansion of the blood flow jet at this point of the aortic anatomy. [Color figure can be viewed in the online issue, which is available at http://wileyonlinelibrary.com.]

During systole, the acceleration phase is stronger and is of shorter duration than deceleration (see Table 3). During diastole, relative pressure is small compared with systole, and is again mainly driven by transient effects, with blood generally accelerated towards the descending aorta by a transient component (5.3 mmHg/m). This transient component shows a slight temporal shift in its temporal waveform alongside the length of the aorta, which can be explained by the effects of pulse wave propagation (see Figure 5).

**Table 3:** Transient Pressure Indexes in Healthy (Mean ± Standard Deviation, n = 9) and Selected Patients Compared to Relevant Indexes of Cardiac Performance: Systemic Systolic and Diastolic Blood Pressure (BP), and Left Ventricular Ejection Fraction (LVEF) and Heart Rate (HR)^a^

	maxP_t_ (mmHg/m)	+T_Pt_ (ms)	minP_t_ (mmHg/m)	−T_Pt_ (ms)	Syst. BP (mmHg)	Diast. BP (mmHg)	LVEF (%)	HR (bpm)
Healthy	64.5 ± 11.7	149 ± 56	−42.7 ± 9.0	235 ± 87	120 ± 26	81 ± 26	60 ± 6	59 ± 6
BAV	49.0	108	−28.7	283	134	83	70%	55
AoD	51.0	121	−32.0	232	136	78	63%	61
MFS	42.8	122	−20.8	266	129	74	63%	64

a maxP_t_ and minP_t_ are the maximum and minimum values respectively of the transient component of the pressure gradient in a length of 270 mm (from the mitral valve, see Figure 2). +T_Pt_ and -T_Pt_ are the duration of the positive and negative two phases of the transient component of pressure (see Figure 4).

Results are consistent with expected characteristics of convective pressure which is positive and large when flow changes direction or when the jet of flow narrows (SV and Arch in Figure 5), negative when the jet of flow expands in diameter (AA1 in Figure 5), and small in straight segments of the vessels (DA2 and DA3 in Figure 5). Further quantification is provided in Supplemental Figure S4.

The viscous component is very small compared with the other components, in keeping with the high Womersley number typical of aortic flows [20]. The highest values of viscous dissipation occur at the SV and the early ascending aorta (see Figure 5), where the interaction between the ejection jet and the vortical flow at the SV occur.

### Patients

Patients typically showed smaller values of transient pressure, with faster acceleration and slower deceleration phase (see Table 3), and localized outstanding values of convective pressure changes (see Figure 3, quantitative comparison in Figure S4, and anatomical detail in Supplemental Figure S5) when compared with volunteers.

In particular, BAV results reveal a marked increase of convective pressure at SV (see Supplemental Figure S4), explained by the narrower valve opening. This case also shows an abrupt pressure change at the end of the arch, explained by a substantial drop in the convective component at the transition from the arch to the descending aorta in systole (see Figure 3), due to a pseudo-coarctation at this site. Finally, a drop in the convective component and small transient acceleration in the AA1 (see Figure 3), colocalized with the region of high vorticity at the centre of the ascending aortic aneurysm, is observed.

AoD shows a complex pattern of convective pressure. There are large changes in pressure at the inlet to the dissected segment at peak systole, end of AA2, reflecting the abrupt increase in aortic dimensions at the point at which the aortic graft re-joins the dissected native aorta. The arch, where the dissection occurs, is quite long in this case, and it produces smaller values of transient and convective components, and an irregular presence of positive and negative fluctuations of convective pressure along its length.

The MFS case has the lowest magnitude of pressure values. Results show a drop in the convective component colocalized with a region of high vorticity at the DA1 during late systole and early diastole. The analysis reveals a “bi-phasic” deceleration of blood, with an early negative peak of relative pressure at 200 ms, and a late peak at 400 ms (mitral valve closure).

## DISCUSSION

This study is, to our knowledge, the first report of the spatiotemporal distribution of relative pressure and its components in the aorta. Results provide initial proof of concept of the capacity of the technique to discriminate, localize, and quantify spatial and temporal pressure characteristics. The localized abrupt changes in pressure identified in patients were the result of geometric abnormalities of the aorta, such as the presence of an aneurysm, a pseudo-coarctation, the inlet of a dissection, or by complex flow features, such as vortical flow.

Particular strengths of our approach include: (i) the use of comprehensive, high spatial and temporal resolution velocity data, not reliant upon traditional assumptions [9], and not limited to 2D views [2,21] (where convective and viscous components can only be reliably computed if flow velocity has no out of plane component); (ii) The use of a finite element computational approach that enables fast, robust and accurate calculation of the entire aortic pressure field, without the need of boundary conditions [6]; (iii) The demonstrated capacity of the method for distinguishing and localising differences between healthy volunteers and patients.

Investigation of central relative pressure was initiated in the 1960s [22] using invasive catheters, a methodology with well-described risks of serious complications, and ionizing radiation. Across the range of noninvasive methods, Doppler-based pressure estimates are used in several clinical situations [21]. Nevertheless, they are operator dependent, require good acoustic windows, are limited to the plane of insonation, and usually rely on simplified equations (Euler equation describing pressure gradients, or Bernoulli equation describing a pressure drop through a narrowing) that make results typically highly sensitive to velocity measurement errors. An alternative approach is central blood pressure estimation from the shape of peripheral pulse contours [23], a technique widely used in research studies [24]. This approach does not provide information about relative pressures, and, as such, should be regarded as complementary.

Transient pressure component originates from the temporal acceleration of blood, which is caused by time-varying forces acting externally on the blood—in particular the vigor and timing of left ventricular ejection and the effect of the compliant aorta. A preliminary definition of cardiac biomarkers based on the magnitude and duration of this component has led to differentiation of patients, see Table 3. The regional analysis of this component reveals a temporal shift of the waveform (see Figure 5), in concordance with the expected propagation of the pressure wave, that suggests new avenues for the computation of compliance indexes.

The convective component, in contrast, is governed principally by the aortic geometry that introduces spatial variations in flow directions. It is the only component accounted by the simplified and modified Bernoulli equations [25], which are used to characterize aortic stenosis and coarctation with Doppler US [26]. This component could characterize the location and timing of aortic complications including aneurysms (drop at AA1 in BAV), or even dissection entry and exit tears (abrupt drop at the entry tear at AA2 in AoD). The convective component has also the potential to characterize the functional degradation of bicuspid valves (high values at SV in BAV).

Results illustrate the presence of higher friction and viscous effects at SV, as could be expected by the presence of vortical flow and high blood velocities. Nevertheless, viscous dissipation was higher in healthy volunteers than in patients, a result that was not expected. One possible explanation is the fact that computation of second order spatial derivatives is very sensitive to data limitations. Another likely cause is the lack of validity of the laminar Newtonian assumption. It should also be noted that 4D flow data, averaged across many heartbeats, attenuates small scale fluctuations (like turbulent or transitional flows). While turbulent flow may not be common in aortic diseases in the absence of severe stenosis, transitional flows are likely to be present in aortic diseases such as BAV, aortic valvular stenosis, and coarctation. In these cases, complementary MRI-based techniques can be used to estimate the fluctuating component of blood flow [28].

Relative pressure based on PC-MRI has been validated using phantoms [9,13] and in vivo animal models [9,12]. Verification results of our finite-element method approach were previously reported [6]. Aortic relative pressure reported in this work agree qualitatively and quantitatively with previous results published in the literature [9,11,13,22]. However, we acknowledge several limitations to the approach described remain. Specifically the decomposition of blood pressure in its three components has not been empirically validated because it is not possible to measure each component separately. Furthermore, segmentation of aortic lumen was performed over an average frame, with no account taken of aortic displacement during the cardiac cycle, thus leading to boundary layer regions not properly included in computations. However, transient and convective components of pressure were computed from sufficient temporal (40 ms of sampling, when the minimum requirement is defined as 44 ms) [2] and spatial (voxel spacing similar to reported validation studies [9,13] resolution. Despite meeting minimum requirements, a temporal sampling of 40 ms removes high frequency characteristics, what is likely to introduce an underestimation of peak values [13,28]. Acquisition by MRI is distributed over time, introducing an additional source of error compared with other modalities, especially at instants of high acceleration. Possible errors due to bulk patient motion during image acquisition have not been accounted for. Changes in blood viscosity due to disease conditions [18] or to temperature have not been considered. The MRI sequence used was prospectively gated, which limited our ability to draw conclusions concerning diastolic events. Finally, we recognize that the ages of healthy subjects differ from those with disease. Our intention was to demonstrate the ability of the technique to characterize a spectrum of appearances across a wide population. Future studies will be needed to define the influence of age and hemodynamic features on relative pressure distributions, and to characterize the relative pressure distributions in large, well characterized, patient cohorts.

## Conclusion

We describe a novel method for the isolation and separate evaluation of the three components of relative pressure. This approach identified a spectrum of patterns across the subjects studied with potential implications for guiding therapy.

## References

[1] Anjum A, Von Allmen R, Greenhalgh R, Powell JT (2012). Explaining the decrease in mortality from abdominal aortic aneurysm rupture. Br J Surg.

[2] Thompson RB, McVeigh ER (2003). Fast measurement of intracardiac pressure differences with 2D breath-hold phase-contrast MRI. Magn Reson Med.

[3] Pasipoularides A (2013). Evaluation of right and left ventricular diastolic filling. J Cardiovasc Transl Res.

[4] Sengupta PP, Pedrizzetti G, Kilner PJ, Kheradvar A, Ebbers T, Tonti G, Fraser AG, Narula J (2012). Emerging trends in CV flow visualization. JACC Cardiovasc Imaging.

[5] Markl M, Kilner PJ, Ebbers T (2011). Comprehensive 4D velocity mapping of the heart and great vessels by cardiovascular magnetic resonance. J Cardiovasc Magn Reson.

[6] Krittian SBS, Lamata P, Michler C, Nordsletten DA, Bock J, Bradley CP, Pitcher A, Kilner PJ, Markl M, Smith NP (2012). A finite-element approach to the direct computation of relative cardiovascular pressure from time-resolved MR velocity data. Med Image Anal.

[7] Yang GZ, Kilner PJ, Wood NB, Underwood SR, Firmin DN (1996). Computation of flow pressure fields from magnetic resonance velocity mapping. Magn Reson Med.

[8] Song SM, Leahy RM, Boyd DP, Brundage BH, Napel S (1994). Determining cardiac velocity fields and intraventricular pressure distribution from a sequence of ultrafast CT cardiac images. IEEE Trans Med Imaging.

[9] Bock J, Frydrychowicz A, Lorenz R, Hirtler D, Barker AJ, Johnson KM, Arnold R, Burkhardt H, Hennig J, Markl M (2011). In vivo noninvasive 4D pressure difference mapping in the human aorta: phantom comparison and application in healthy volunteers and patients. Magn Reson Med.

[10] Ebbers T, Farneback G (2009). Improving computation of cardiovascular relative pressure fields from velocity MRI. J Magn Reson Imaging.

[11] Ebbers T, Wigstrom L, Bolger AF, Engvall J, Karlsson M (2001). Estimation of relative cardiovascular pressures using time-resolved three-dimensional phase contrast MRI. Magn Reson Med.

[12] Lum DP, Johnson KM, Paul RK, Turk AS, Consigny DW, Grinde JR, Mistretta CA, Grist TM (2007). Transstenotic pressure gradients: measurement in swine--retrospectively ECG-gated 3D phase-contrast MR angiography versus endovascular pressure-sensing guidewires. Radiology.

[13] Tyszka JM, Laidlaw DH, Asa JW, Silverman JM (2000). Three-dimensional, time-resolved (4D) relative pressure mapping using magnetic resonance imaging. J Magn Reson Imaging.

[14] Pitcher A, Lamata P, Krittian SB (2013). Towards a comprehensive description of relative aortic pressure: insights from 4D flow CMR. J Cardiovasc Magn Reson.

[15] Markl M, Harloff A, Bley TA, Zaitsev M, Jung B, Weigang E, Langer M, Hennig J, Frydrychowicz A (2007). Time-resolved 3D MR velocity mapping at 3T: improved navigator-gated assessment of vascular anatomy and blood flow. J Magn Reson Imaging.

[16] Yushkevich PA, Piven J, Hazlett HC, Smith RG, Ho S, Gee JC, Gerig G (2006). User-guided 3D active contour segmentation of anatomical structures: significantly improved efficiency and reliability. Neuroimage.

[17] Ebbers T, Wigstrom L, Bolger AF, Wranne B, Karlsson M (2002). Noninvasive measurement of time-varying three-dimensional relative pressure fields within the human heart. J Biomech Eng.

[18] Lowe GDO, Lee AJ, Rumley A, Price JF, Fowkes FGR (1997). Blood viscosity and risk of cardiovascular events: The Edinburgh Artery Study. Br J Haematol.

[19] Lee T, Kashyap R, Chu C (1994). Building skeleton models via 3-d medial surface axis thinning algorithms. CVGIP Graph Model Image Process.

[20] Stalder AF, Frydrychowicz A, Russe MF, Korvink JG, Hennig J, Li K, Markl M (2011). Assessment of flow instabilities in the healthy aorta using flow-sensitive MRI. J Magn Reson Imag.

[21] Yotti R, Bermejo J, Benito Y (2011). Noninvasive estimation of the rate of relaxation by the analysis of intraventricular pressure gradients. Circ Cardiovasc Imaging.

[22] McDonald DA (1955). The relation of pulsatile pressure to flow in arteries. J Physiol Lond.

[23] Karamanoglu M, O'Rourke MF, Avolio AP, Kelly RP (1993). An analysis of the relationship between central aortic and peripheral upper limb pressure waves in man. Eur Heart J.

[24] Hope SA, Meredith IT, Cameron JD (2008). Arterial transfer functions and the reconstruction of central aortic waveforms: myths, controversies and misconceptions. J Hypertens.

[25] Pasipoularides A, Katz AM (1990). Clinical assesment of ventricular ejection dynamics with and without outflow obstruction. J Am Coll Cardiol.

[26] Tan J-L, Babu-Narayan SV, Henein MY, Mullen M, Li W (2005). Doppler echocardiographic profile and indexes in the evaluation of aortic coarctation in patients before and after stenting. J Am Coll Cardiol.

[27] Dyverfeldt P, Kvitting J-PE, Sigfridsson A, Engvall J, Bolger AF, Ebbers T (2008). Assessment of fluctuating velocities in disturbed cardiovascular blood flow: in vivo feasibility of generalized phase-contrast MRI. J Magn Reson Imaging.

[28] Firstenberg MS, Vandervoort PM, Greenberg NL, Smedira NG, McCarthy PM, Garcia MJ, Thomas JD (2000). Noninvasive estimation of transmitral pressure drop across the normal mitral valve in humans: importance of convective and inertial forces during left ventricular filling. J Am Coll Cardiol.

