# Supplementary material for: Aortic Relative Pressure Components Derived from Four-Dimensional Flow Cardiovascular Magnetic Resonance
Source: Magn Reson Med. 2013 Nov 18;72(4):1162–9. doi: 10.1002/mrm.25015 (PMC4024466; doi:10.1002/mrm.25015)
Supplement: Supplementary file 1 — Supporting Information [file mrm0072-1162-sd1.doc]

**Supplemental material**


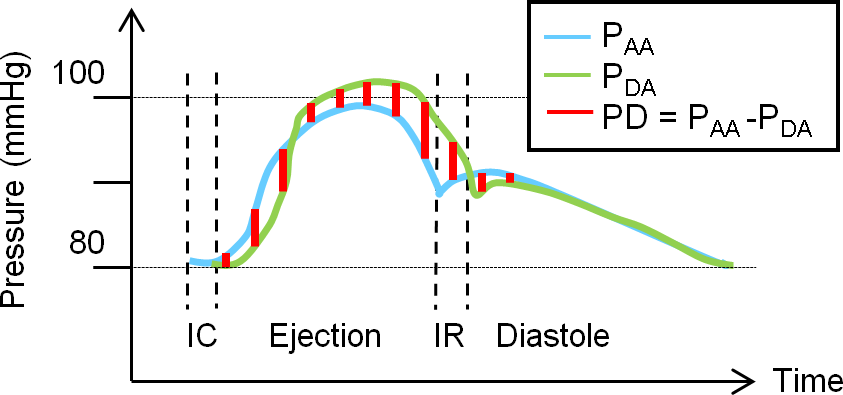


Fig.S1: Illustration of the concept of *relative* pressure, or pressure difference (PD) between a point, (at the ascending aorta in the graph, P_AA_), to a reference location (in this case at the descending aorta, P_DA_). The analysis of the blood flow velocity, by solving the Navier-Stokes equation, enables the computation of relative pressure with respect to a reference point in the field of view (PD), not the computation of the *absolute* pressure with respect to the atmospheric pressure (P_AA_ or P_DA_).


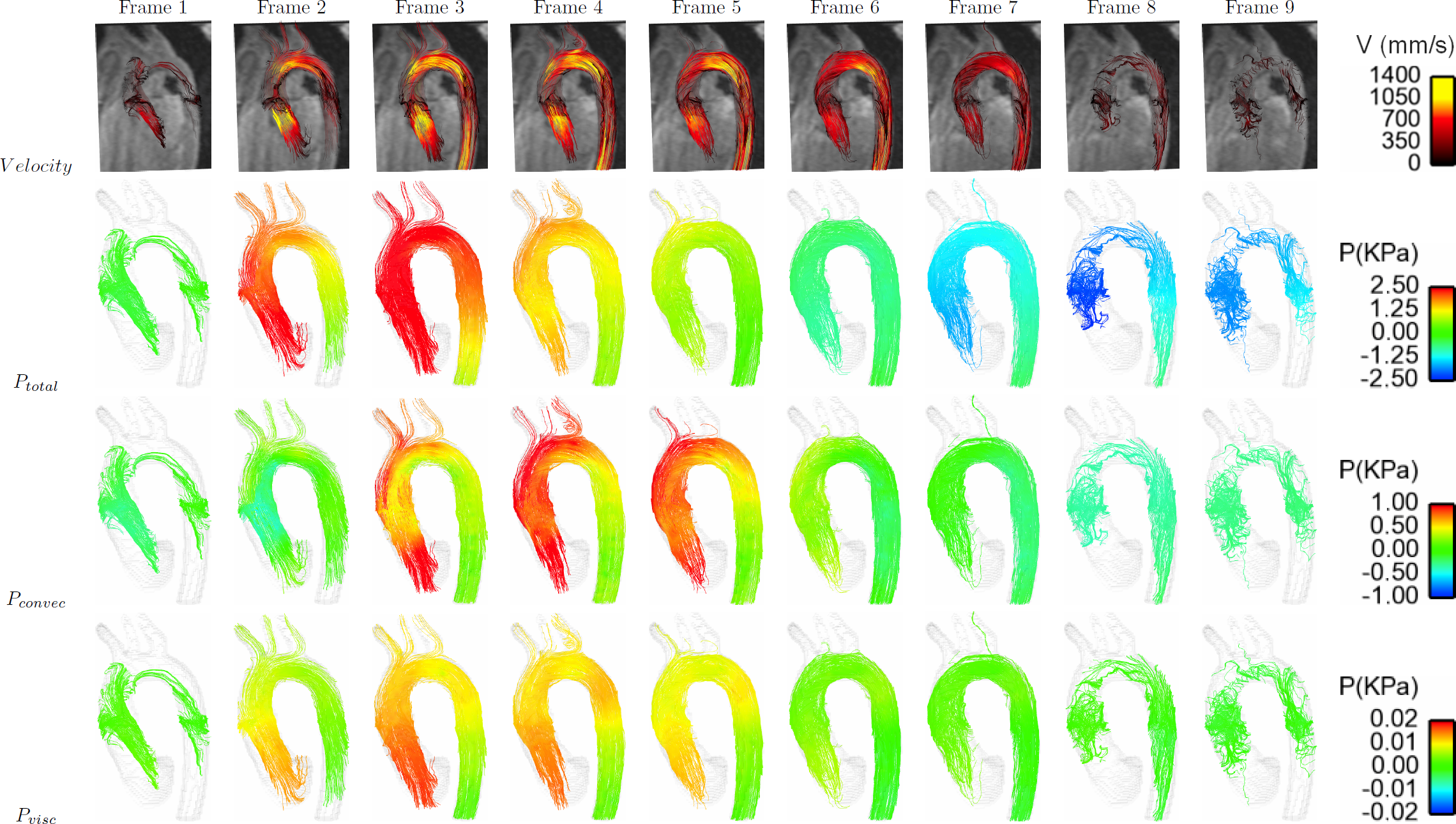


Fig.S2: Velocity and pressure differences (total, convective and viscous) in a healty volunteer (HV2) during the first 9 frames of the PC-MRI sequence corresponding to systole. The same streamlines in all four series are coloured according to the velocity or pressure magnitude.


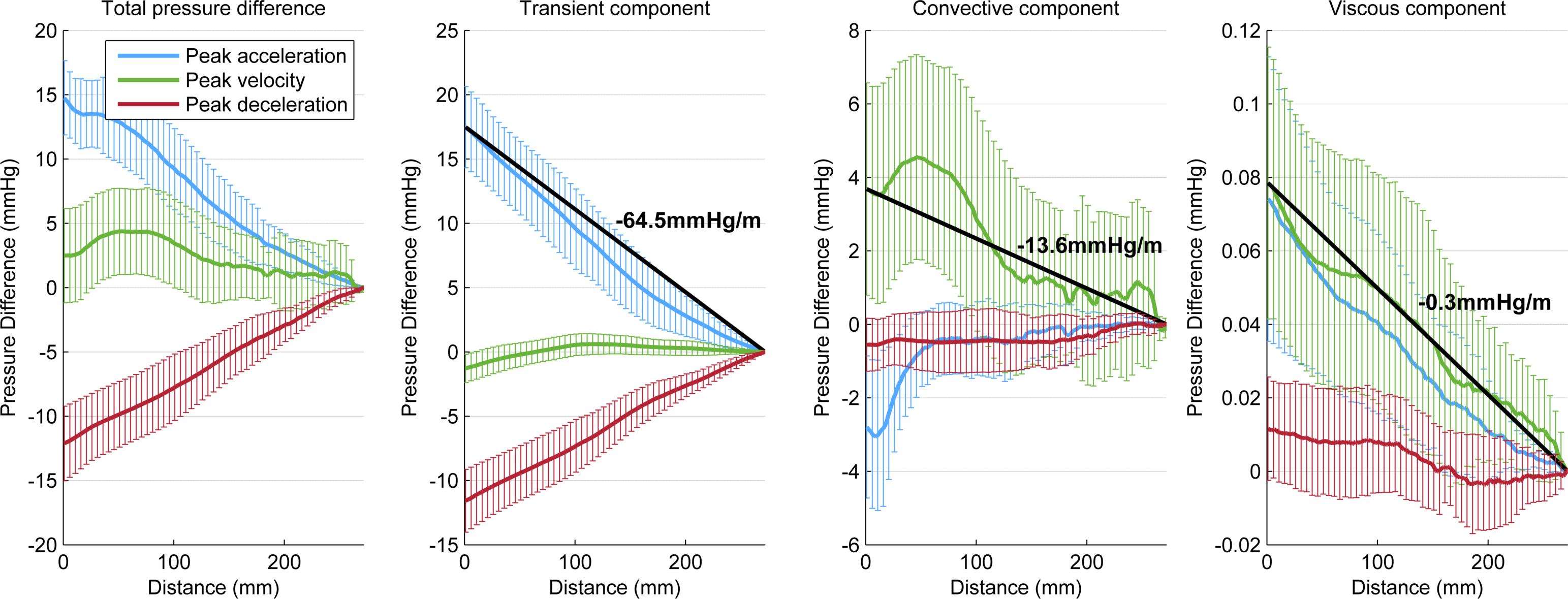


Fig.S3: Average spatial evolution of the aortic relative pressure in the nine healthy subjects at three instants of systole. Distances are measured from the aortic valve plane (plane 1 in Fig.2, main manuscript) until the point at 270mm (the longest distance of all nine cases). Confidence intervals indicate mean +/- 1 std. Black lines illustrate the average pressure gradient at peak instants of each of the three components (with text reporting values).


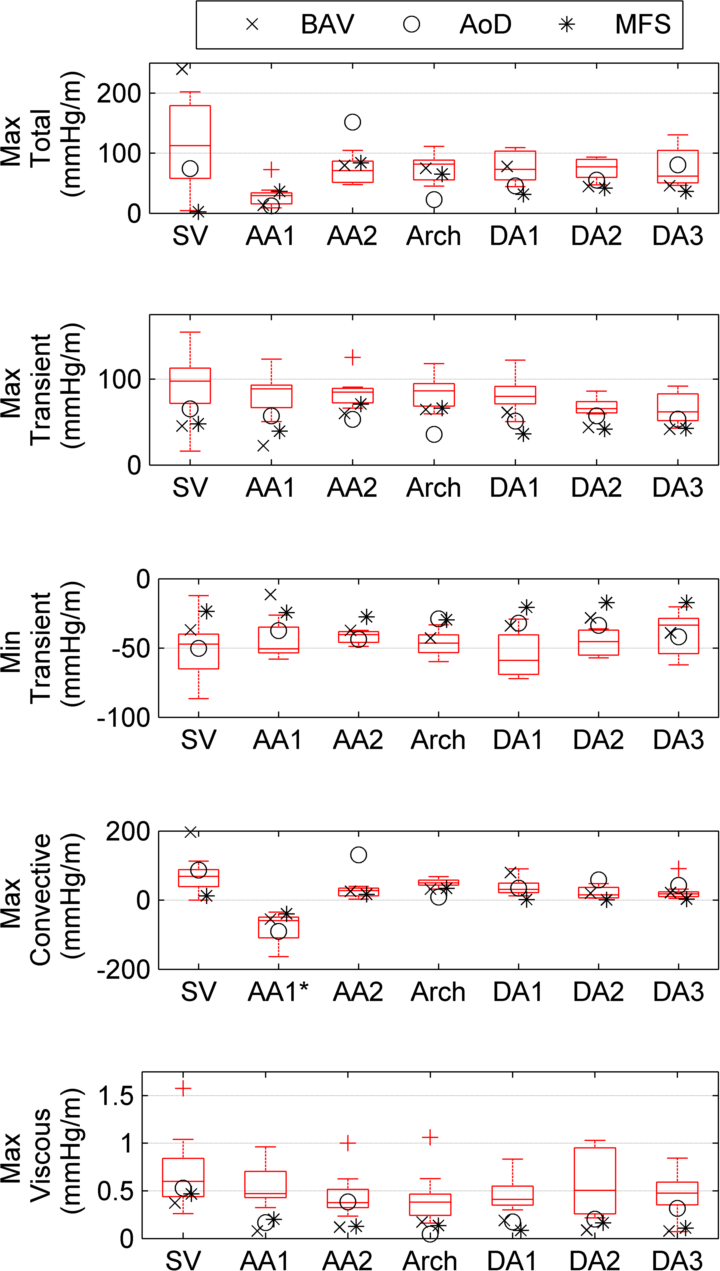


Fig.S4: Comparative regional analysis of peak values (max or min) of pressure gradient (PG, see definition in Fig.2, main manuscript) in the aorta, and of its three components. *: min peak at AA1 for the convective component due to its negative nature (see Fig.5, main manuscript).


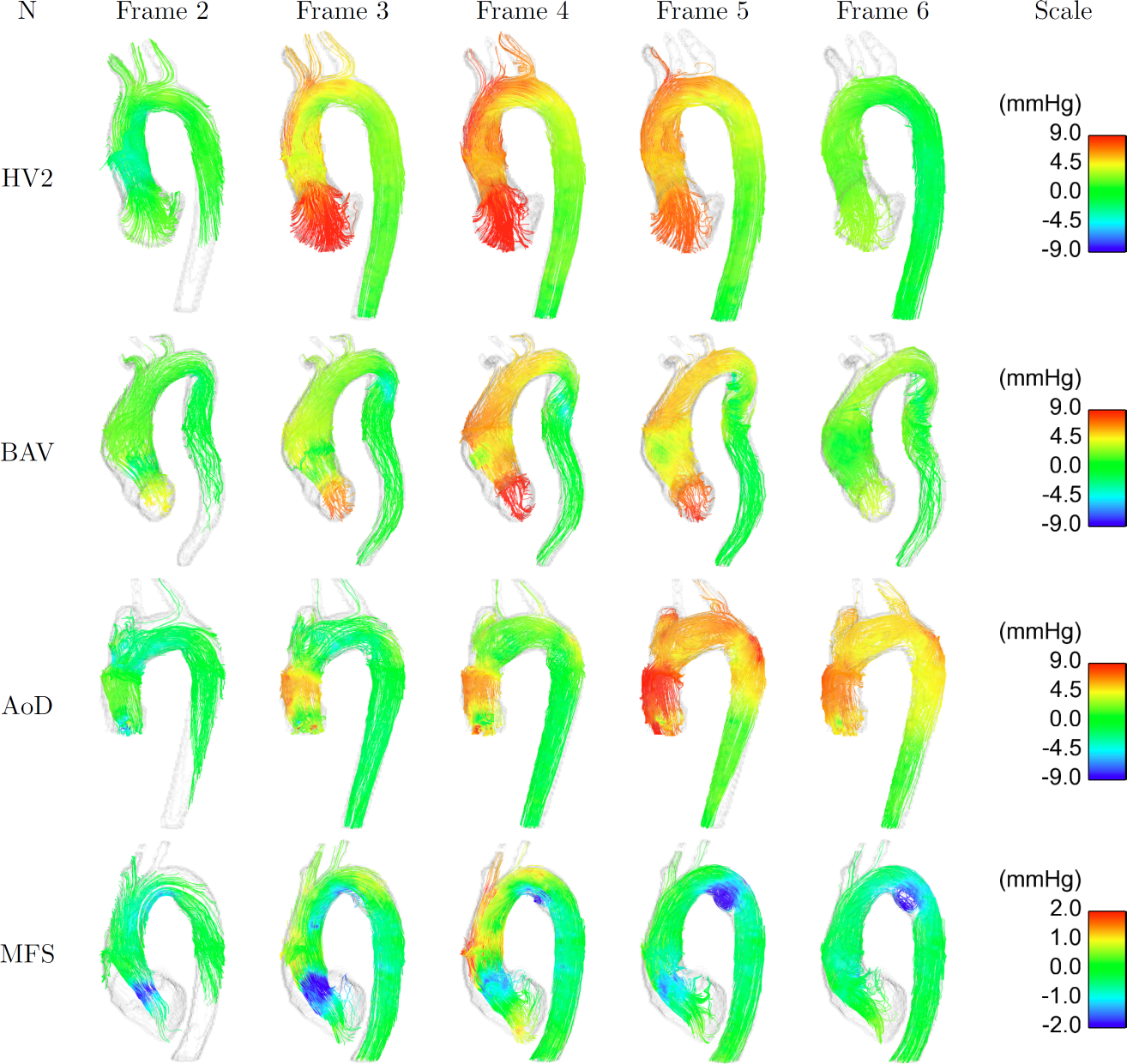


Fig.S5: Anatomical inspection of the convective pressure, showing velocity streamlines coloured by pressure, at different systolic frames. MFS shows vortexes characterised by a drop of pressure, and a pseudo-coarctation in BAV shows an abrupt drop at the beginning of DA1.
